# Elevated urine IL-10 concentrations associate with *Escherichia coli* persistence in older patients susceptible to recurrent urinary tract infections

**DOI:** 10.1186/s12979-019-0156-9

**Published:** 2019-07-11

**Authors:** Lauren K. L. Drage, Wendy Robson, Catherine Mowbray, Ased Ali, John D. Perry, Katherine E. Walton, Christopher Harding, Robert Pickard, Judith Hall, Phillip D. Aldridge

**Affiliations:** 1 Centre for Bacterial Cell Biology, Baddiley-Clark Building, Newcastle University, Newcastle upon Tyne, UK; 2 Institute for Cell and Molecular Biosciences, Newcastle University, Newcastle upon Tyne, NE2 4AH UK; 3 Urology Department, Freeman Hospital, Newcastle upon Tyne Hospitals NHS Foundation Trust, Newcastle upon Tyne, UK; 4 Institute for Cellular Medicine, Newcastle University, Newcastle upon Tyne, UK; 5 Microbiology Department, Freeman Hospital, Newcastle upon Tyne Hospitals NHS Foundation Trust, Newcastle upon Tyne, UK; 6 Present Address: GlaxoSmithKline, Gunnels Wood Road, Stevenage, SG1 2NY UK; 7 Present Address: Department of Urology, Pinderfields Hospital, Wakefield, WF1 4DG UK

**Keywords:** Urinary tract infection, *Escherichia coli*, Cytokines, Ageing, Antibiotics

## Abstract

**Background:**

Age is a significant risk factor for recurrent urinary tract (rUTI) infections, but the clinical picture is often confused in older patients who also present with asymptomatic bacteriuria (ASB). Yet, how bacteriuria establishes in such patients and the factors underpinning and/or driving symptomatic UTI episodes are still not understood. To explore this further a pilot study was completed in which 30 male and female community based older patients (mean age 75y) presenting clinically with ASB / rUTIs and 15 control volunteers (72y) were recruited and monitored for up to 6 months. During this period symptomatic UTI episodes were recorded and urines collected for urinary cytokine and uropathogenic *Escherichia coli* (UPEC) analyses.

**Results:**

Eighty-six per cent of patients carried *E. coli* (10^2^ ≥ 10^5^ CFU/ml urine) at some point throughout the study and molecular typing identified 26 different *E. coli* strains in total. Analyses of urine samples for ten different cytokines identified substantial patient variability. However, when examined longitudinally the pro-inflammatory markers, IL-1 and IL-8, and the anti-inflammatory markers, IL-5 and IL-10, were significantly different in the patient urines compared to those of the controls (*P* < 0.0001). Furthermore, analysing the cytokine data of the rUTI susceptible cohort in relation to *E. coli* carriage, showed the mean IL-10 concentration to be significantly elevated (*P* = 0.04), in patients displaying *E. coli* numbers ≥10^5^ CFU/ml.

**Conclusions:**

These pilot study data suggest that bacteriuria, characteristic of older rUTI patients, is associated with an immune homeostasis in the urinary tract involving the synthesis and activities of the pro and anti-inflammatory cytokines IL-1, IL-5, IL-8 and IL-10. Data also suggests a role for IL-10 in regulating bacterial persistence.

## Background

Recurrent urinary tract infections (rUTI) are a major clinical problem, particularly in older age groups, impacting significantly on patient well-being and global healthcare systems [1, 2]. Such infections are classified as complicated or uncomplicated, depending on the presence or absence of structural or functional abnormalities of the urinary tract, but are linked to the persistent breaching of the host innate urinary tract defences by uropathogenic bacteria [3]. In treating rUTIs, patients are often prescribed repeated short-term antibiotic treatments or receive long-term, low-dose prophylaxis therapies. Continuous use of these antimicrobial agents has been shown to impact UTI frequency, but at the cost of bacterial resistance, which is a major public health concern [4–6].

Age is a significant risk factor for rUTI [2, 7]. However, the clinical picture is frequently confused by older patients who present with significant bacteriuria, but without the symptoms or other adverse effects associated with an UTI [2]. This harmless condition is termed asymptomatic bacteriuria (ASB) and it has been suggested that the microbial strains colonising the urinary tract, and associated with bacteriuria evolve from their virulent predecessors [8, 9]. Still, how bacteriuria establishes, the level of host-pathogen communication that occurs and the factors underpinning and/or driving symptomatic UTI episodes are not well understood.

Asymptomatic bacteriuria would not normally be treated with antibiotics [7, 10]. Yet many older patients, particularly those with cognitive impairments where history-taking is clinically challenging, are often subjected to frequent, but needless antibiotic treatment regimens that do not cure or eradicate the bacteriuria, but actually drive microbial resistance [11]. However, the clinical dilemma is considerable because leaving a suspected UTI untreated in such patients may allow the infection to progress resulting in pyelonephritis, septicaemia and in some cases death [2, 12], but if treated unnecessarily can predispose individuals to opportunistic infections such as *Clostridium difficile* antibiotic induced diarrhoea [11]. This conundrum illustrates the need for further investigations in older patient groups, specifically focussing on host-microbial interactions during periods of bacteriuria or asymptomatic carriage and infection.

Asymptomatic bacteriuria is associated with a range of bacterial species including the Enterobacteriaceae *Escherichia coli*, *Klebsiella pneumoniae and Proteus mirabilis* and Gram-positive bacteria including *Enterococcus* [3, 7]. Of the species isolated from urine *E. coli* is the most common identified agent with uropathogenic *E.coli* or UPEC linked to > 75% of all reported UTIs [2, 3]. Protection of the lower urinary tract from microbial assault is mediated through innate defence mechanisms that include physical factors such as urine pH, ionic composition and flow. These, in conjunction with innate immune responses characterised by the constitutive or inducible synthesis of urothelial host defence molecules including antimicrobial agents, chemokines and cytokines, function to prevent infection [13]. In healthy individuals these factors work rapidly and collectively to contain, and eliminate uropathogens [13, 14] from the urinary tract, but in older groups, especially those with incomplete bladder emptying, and comparable groups including those afflicted by neural or spinal pathologies, a bacterial presence described as ASB is common.

In establishing diagnostic tools to differentiate ASB from an UTI the focus has been on urinary cytokines and chemokines, which are easily measured and presumed to reflect the immune response of the urinary tract. To date only IL-6, a pro-inflammatory cytokine, has been nominated as a potential biomarker of infection in older patients, but the diagnostic thresholds remain confusing [15–17]. To address this combinations of IL-6 and, for example, leukocyte esterase have been suggested to help improve diagnosis [17]. The key aim of this pilot study was to further understand host-bacteriuria interactions that may help facilitate the development of robust diagnostics that direct appropriate antibiotic treatment regimens. The study was designed to allow the host cytokine response of a mixed population of 30 male and female community-based patients aged 65+ years of age, presenting with a clinical history of uncomplicated rUTIs, to be examined over a 6-month study period. Urine was collected at 14-day intervals, in an unbiased manner and irrespective of UTI status or clinical treatments, with the objectives of exploring host-bacterial, specifically *E. coli,* interactions and identifying potential bacterial persistence and urine infection biomarkers.

## Results

### Study population characteristics and baseline data

**Fig. 1:**
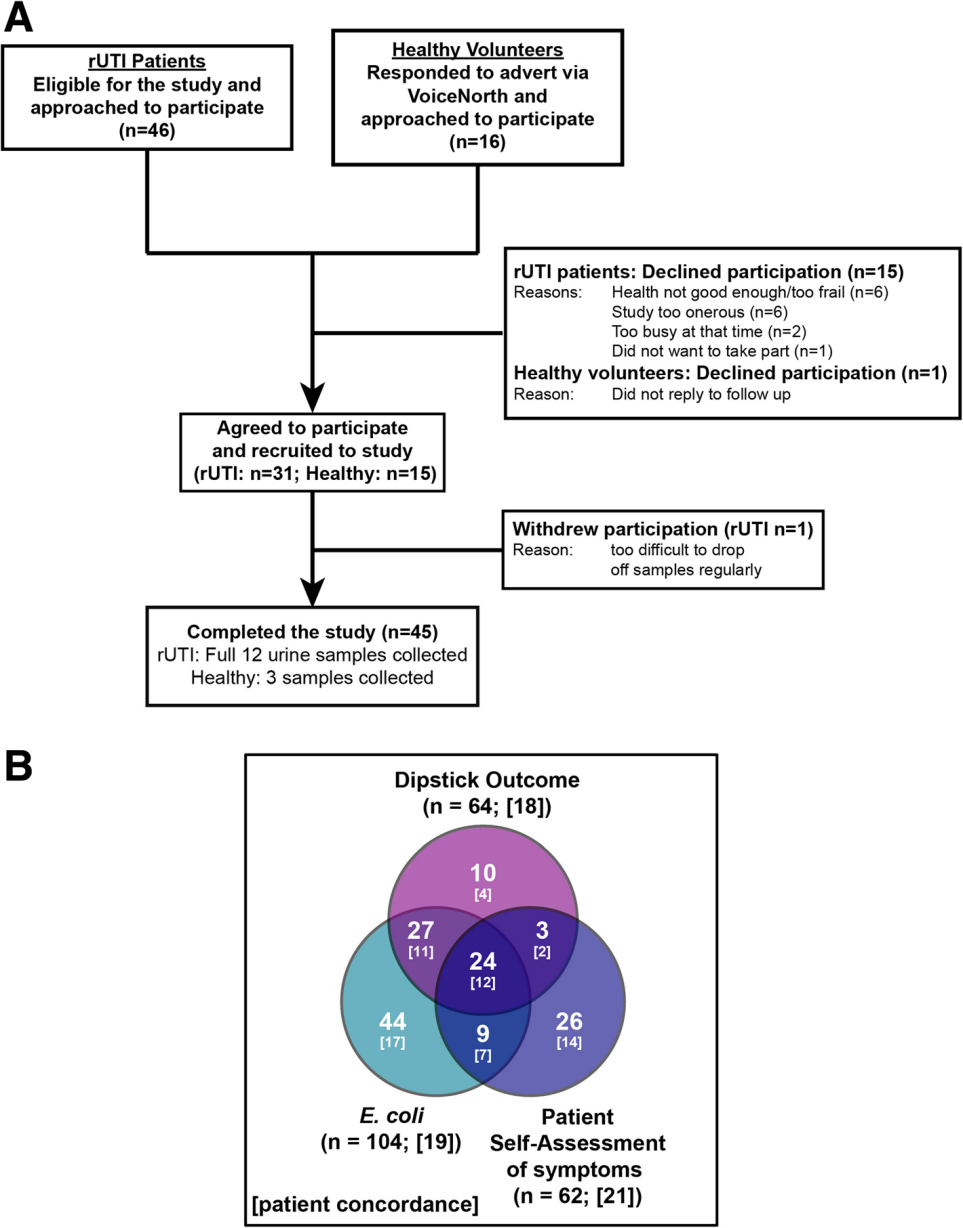
Recruitment Pathway for study and **a** Pilot study summary. Consort diagram of the study recruitment pathway. **b** Venn diagram of positive outcome data for the urine dipstick test (pink), patient self-assessment of symptoms (blue) and *E. coli* isolation at ≥10^5^ CFU/ml (green). [ ] = patient number

The target population for this 6-month study was mixed, males and females, aged 65 years plus that were community dwelling. Recurrent UTI patients were selected specifically because of their clinical history of uncomplicated rUTIs, all attended an UTI out-patients clinic at Freeman Hospital, Newcastle upon Tyne, and lack of co-morbidities. The cohort included 23 females and 7 males with an average age of 74.0 and 76.7 years, respectively (Table 1 and Fig. 1a, ANOVA *P* = 0.26). Patients with structural abnormalities were included if clinical records indicated no evidence of a functional defect in the urinary tract impeding bladder voidance. The study group included 11 patients with either vaginal prolapse (Female *n* = 5), enlarged prostate (Male *n* = 3), urethral stenosis (*n* = 2) or phimosis (*n* = 1). Diabetic patients or females using either topical or systemic oestrogen were also not excluded (Table 1).

**Table 1:** rUTI Demographic patient data

	Female (*n* = 23)	Male (*n* = 7)	ANOVA *P*-value
Age	74.0 ± 5.5	76.7 ± 5.3	0.26
UTI History			
Confirmed^a^	5.4 ± 2.7	3.1 ± 1.1	
Urinary Tract Physiology			
Normal	16	3	
Abnormal^b^	7	4	
Diabetic	4	1	
Oestrogen Use			
Topical	8	N/A	
Systemic	1	N/A	
Structural Abnormality^b^			
Female			
Vaginal prolapse	5		
Urethral prolapse	2		
Urethral stenosis	2		
Male			
Enlarged prostate		3	
Phimosis		1	
Trabeculated bladder		2	

^a^Based on clinical records that stated acute UTI (see Methods)

^b^Patients were included with one or more abnormalities

The control healthy volunteers were selected because they had no clinical history of UTIs over the previous 3 years or longer (Fig. 1a). Five female and 10 male volunteers were recruited with an average age of 68.0 and 73.9 years, respectively. The average age of the group was not significantly different from the rUTI participants (ANOVA *P* = 0.16), although the average age of the female control cohort was younger, statistically, when compared to their rUTI counterparts (ANOVA *P* = 0.02).

### Correlation of diagnostic criteria

A combination of criteria are used to establish an UTI diagnosis [18, 19]. These include assessment of clinical symptoms, the use of a dipstick assay, where a urine positive for nitrite and leukocyte esterase indicates a high probability of an UTI, and microbiological investigation of a urine sample including colony counts [18]. However, even using all these criteria, it is recognised that diagnosis of an UTI as opposed to ASB is challenging and particularly within older populations [18, 19]. While it is acknowledged that a number of different uropathogens can cause UTIs this study focussed on the most common uropathogen, *E. coli* and counts of ≥10^5^ CFU/ml were defined as a significant bacteriuria.

Over the six-month study period a total of 360 urine samples relating to the 30 patients (12 urines per patient) in the rUTI cohort were collected and 1080 measurements performed. Data analyses focussed on the number of symptomatic episodes experienced and self-reported via the UTISA questionnaire (S) [20], positive urine dipstick outcomes (D) and *E. coli* culture numbers per urine sample (E). These measurements were grouped (S + D + E) to reflect 360 patient data points (30 patients × 12 sampling points) and analysed via the Venn diagram shown in Fig. 1b. Only 62/360 data points identified self-reported positive symptomatic UTI episodes (21 patients), 64/360 reflected positive urine dipstick results (18 patients) and 104/360 related to urinary *E. coli* numbers ≥10^5^ CFU/ml (19 patients). There were only 24 data points (12 patients) when all three parameters (symptoms, dipstick and *E. coli* numbers ≥10^5^ CFU/ml) were indicative of an UTI.

As expected from the inclusion criteria no control volunteers reported any symptoms, although one returned three dipstick positive outcomes that were not supported by significant bacteriuria. One further control subject showed significant loads of *E. coli* (≥ 10^5^ CFU/ml), but no positive dipstick result and undetectable cytokine levels (data not shown).

### Strain shifts and antibiotic therapy

**Fig. 2:**
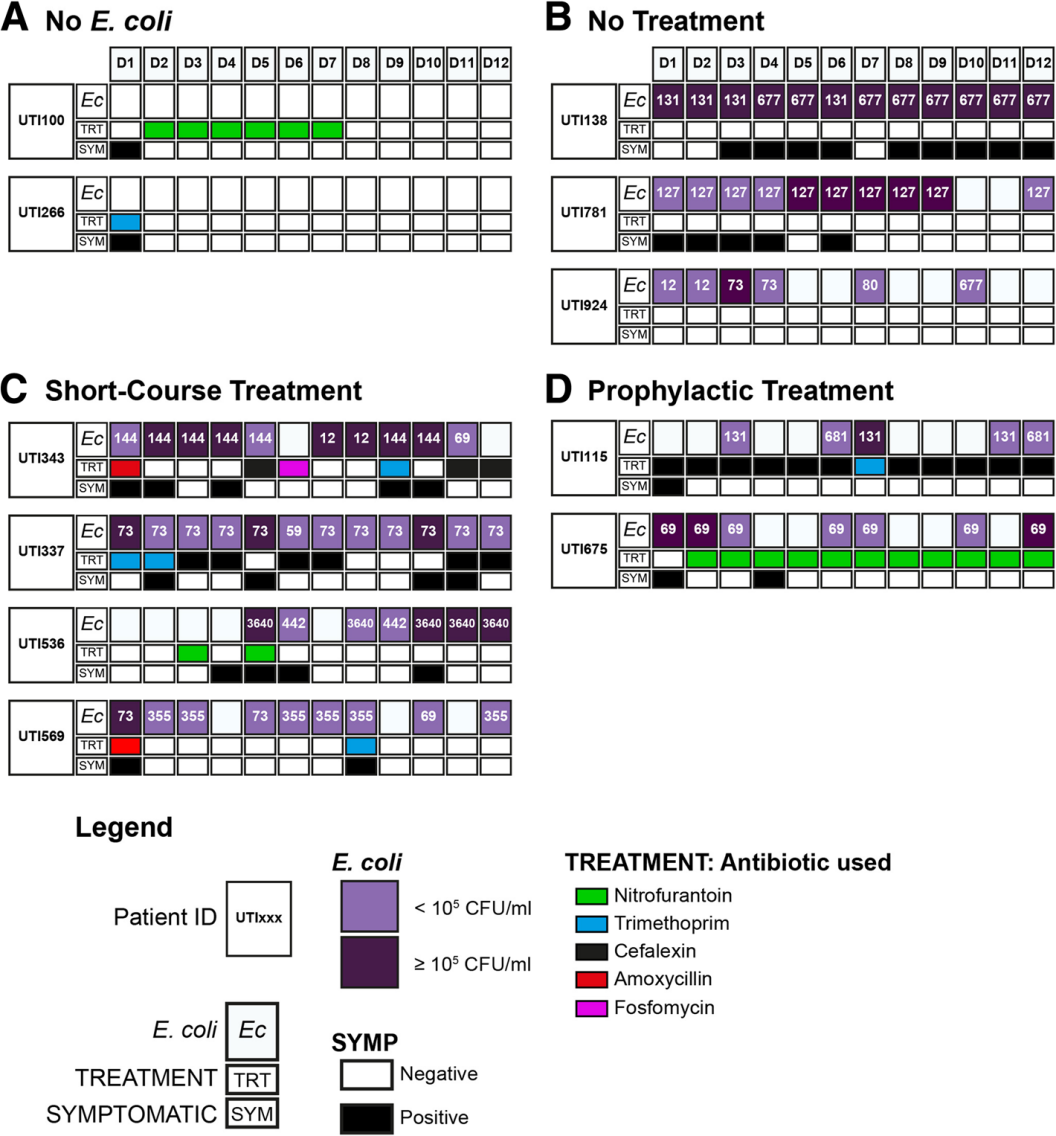
Schematic representation of study data for seven rUTI patients. *E. coli* loads, antibiotic treatments and symptom reports are defined in the attached key. The numbers in the *E. coli* boxes represent the sequence types derived from the MLST analysis. **a** Case examples of patients who received antibiotics and did not present with urinary *E. coli* loads during the study. **b** Case examples of patients who did not receive antibiotic treatments but displayed urinary *E. coli* loads throughout the study. **c** Case examples of patients on short-term (3–7 day courses) of antibiotics. **d** Two case examples of patients on prophylactic antibiotics and where consistent *E. coli* loads were observed

Microbiological analyses of each urine sample revealed that 86% of the rUTI patients (26/30) had a positive urine *E. coli* culture, CFU ranging between 10^2^ to ≥10^5^ CFU/ml, at least once during the study period. Two of the three patients whose urines were identified as being negative for *E. coli* were in receipt of antibiotics at some point during the study period (Fig. 2a). In fact, antibiotic treatment amongst all rUTI patients was common and shown to be beneficial in reducing *E. coli* loads but was also associated with *E. coli* persistence. This is illustrated particularly in patients UTI675 and UTI115 where prophylactic antibiotic therapies reduced *E. coli* loads to below diagnostically significant thresholds, but did not eliminate colonisation or prevent recurrence (Fig. 2d). It was noted however, that no antibiotic treatment during the 6-month study was also associated with bacterial persistence (Fig. 2b).

*E. coli* is a versatile bacterial species that exhibits a range of commensal and pathogenic interactions within the human population [21, 22] with strain typing, including sero- and molecular typing, utilised to characterise the different strains [21]. All *E. coli* strains, however, fit within seven phylogenetic clades (A, B1, B2, D, E and F) with uropathogenic *E. coli* (UPEC) predominantly associated with clade B2 [21]. Molecular typing [23] of the *E. coli* strains isolated from the rUTI patient cohort indicated that some patients, e.g. UTI675, retained strains with the same sequence type (ST) despite antibiotic treatment (Fig. 2d). Examples of shifts to an alternative *E. coli* strain or invasion by a new sequence type of between 10^2^ to < 10^5^ CFU/ml urine were also evident. This is illustrated particularly by the detection of strains ST59 in patient UTI337, ST73 and 69 in UTI569 (Fig. 2c) and ST12, 80 and 677 in UTI924 (Fig. 2b). Overall 26 unique STs were identified and the majority were from phylogenetic clade B2, with ST73 being the most frequent (Table 2).

**Table 2:** *E. coli* sequence types (ST) isolated and sorted by isolation statistics

Phylogroup^a^	Sequence Type	Frequency Isolated^b^	Patients^c^
B1	677	9	2
	3640	5	1
	442	2	1
	602	1	1
	1571	1	1
B2	73	54	9
	12	15	4
	127	11	2
	420	11	1
	131	10	3
	404	8	1
	144	7	1
	95	6	2
	355	6	1
	91	5	1
	625	2	1
	681	2	1
	80	1	1
	421	1	1
	583	1	1
D	69	12	6
	362	2	1
	38	1	1
E	335	1	1
F	354	9	1
	59	1	1

^a^Historical phylogroups or clades of the *E. coli* species utilized across the microbiology community [21]

^b^Isolation frequency based on how many times each sequence type (ST) was identified in the strain collection

^c^Number of patients harbouring specific STs

### Cytokine profiles

**Fig. 3:**
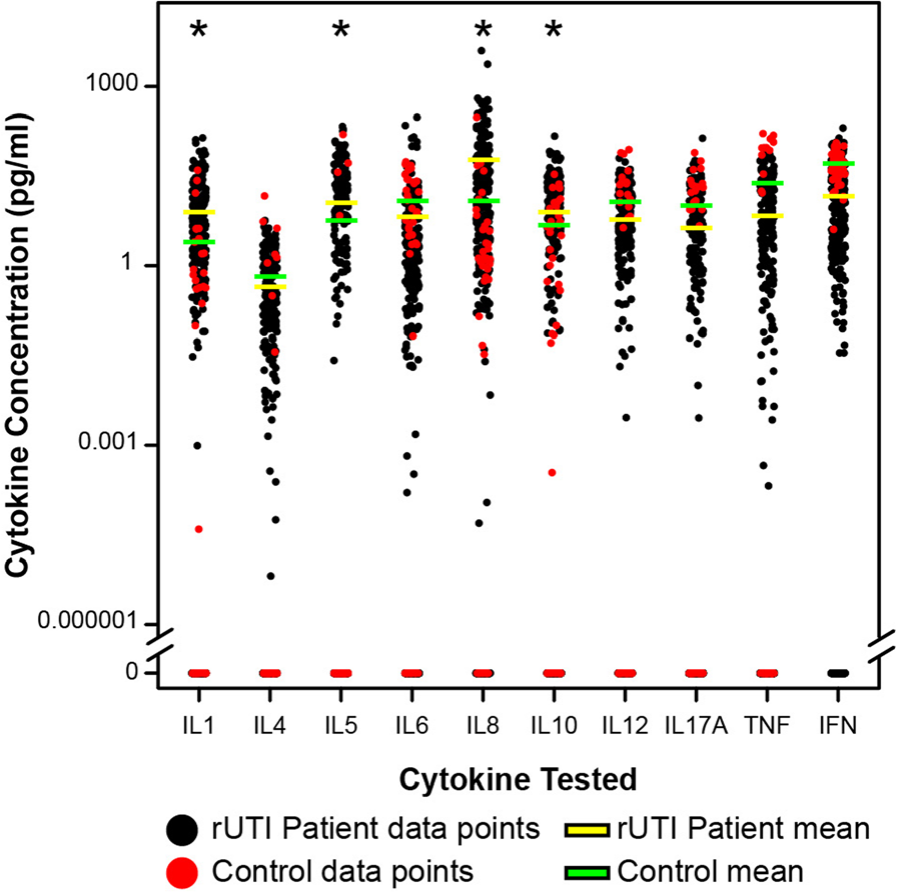
Cytokine concentrations of the 360 rUTI patient (black dots) and 45 control (red dots) urine samples. Mean values are shown for patients with rUTI (yellow line (see key)) and urine from controls (green line). The plot uses a log scale on the y-axis to capture all data points with samples showing undetectable levels of each cytokine in the bottom panel. Only IL-1β, IL-5, IL-8 and IL-10 exhibited significantly elevated levels in patients compared to control samples: * ANOVA *P* < 0.05

To identify potential biomarkers associated with *E. coli* carriage and infection, the urine concentrations of an array of pro and anti-inflammatory cytokines, linked to UTIs and including IL-1β, IL-4, IL-5, IL-6, IL-8, IL-10, IL-12, IL-17A, TNFα and IFNγ were measured [17, 24–30]. Quantification of these ten innate effectors in rUTI patient and control urines highlighted substantial variability, but statistical analyses of the longitudinal data supported the mean concentrations of four cytokines, the pro-inflammatory markers, IL-1β and IL-8, and the anti-inflammatory markers, IL-5 and IL-10, to be significantly elevated in the rUTI patient cohort compared to the controls (Fig. 3, *P* < 0.001).

**Fig. 4:**
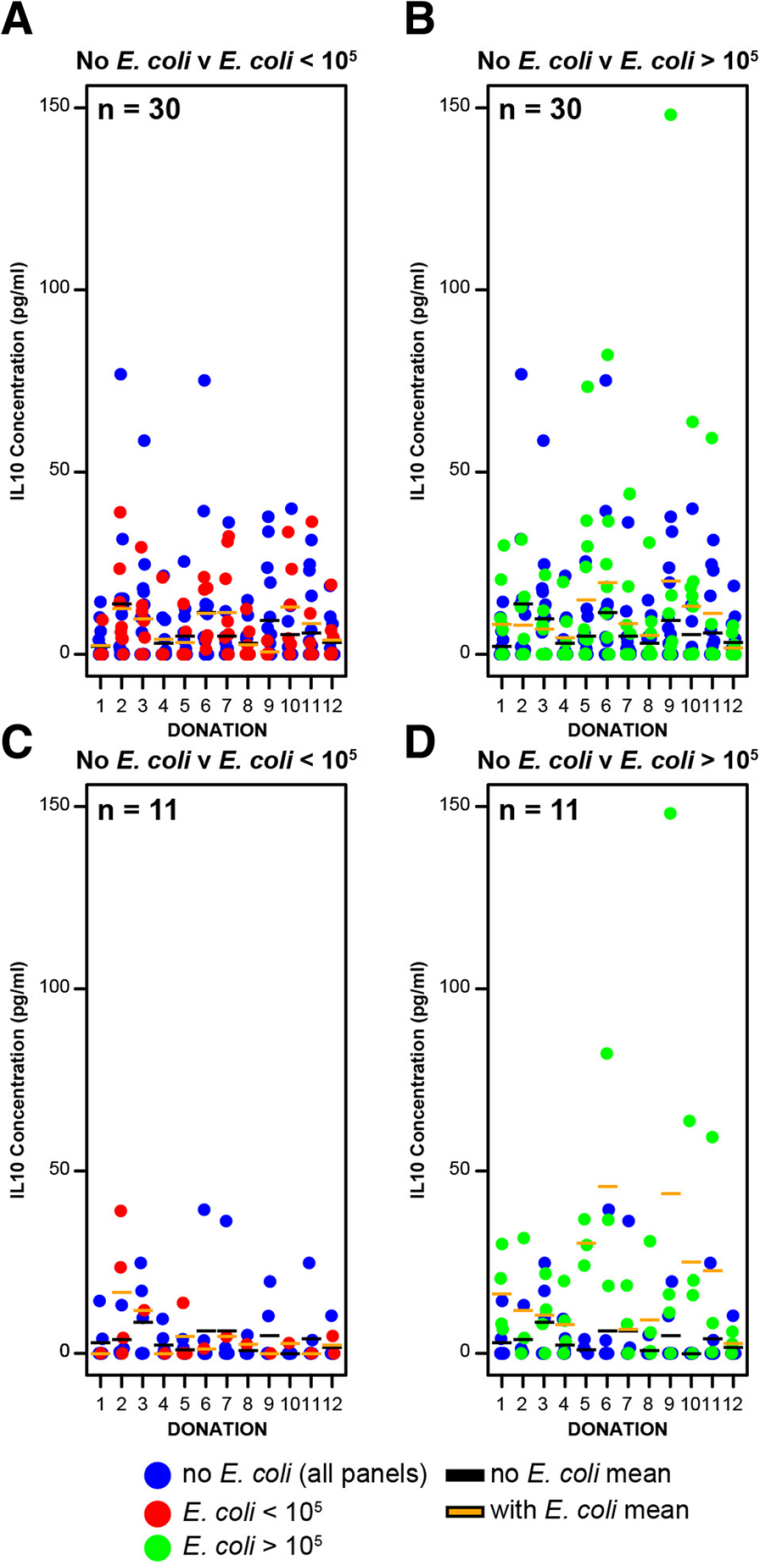
Urinary IL-10 concentrations of rUTI patients. X-axis reflects the longitudinal aspect of the study. In all four panels IL-10 concentrations from patients with no detectable *E. coli* (no *E. coli*) are shown as blue dots (see key). Mean values are shown for no *E. coli* in all panels as a black line. **a** no *E. coli* to < 10^5^ CFU/ml *E. coli* (red dots; mean value: orange line) **b** no *E. coli* to > 10^5^ CFU/ml *E. coli* was observed (green dots; mean value: orange line). **c** and **d** Focus on 11 rUTI patients with normal urinary tract physiology and/or no complicated medical history. Colours of dots, lines and comparisons are the same as in (**a**) and (**b**) and highlighted in the figure key. Statistical analysis and average concentrations of the data presented are found in Table 3

Of the 360 rUTI patient urines analysed, 184 were positive for *E. coli* of which 80 displayed > 10^2^ <  10^5^ CFU/ml, and 104 samples registered ≥10^5^ CFU/ml, which, clinically, is defined as ‘significant bacteriuria’ or indicative of an UTI [31]. Comparing urine samples that were negative for *E. coli* to those that harboured *E. coli* at counts of > 10^2^ CFU/ml revealed no significant differences in the urinary IL-1β, IL-5, IL-6, IL-8 and IL-10 cytokine concentrations (Table 3) although statistically these data did suggest a link between IL-10 and the presence of *E. coli* (*P* = 0.08). However, when the cytokine concentrations of rUTI urine samples negative for *E. coli* were compared to those containing ≥10^5^ CFU/ml bacteria a significant increase in IL-10 (6.45 ± 12.26 pg/ml versus 10.57 ± 20.85 pg/ml [*P* = 0.04]) (Fig. 4 and Table 4), was observed. Presented longitudinally these urine data support significantly elevated IL-10 concentrations in rUTI patients carrying ≥10^5^ CFU/ml *E. coli* loads (Fig. 4a and b). The impact of antibiotic treatment with respect to IL-10 showed no statistical significance (Table 4).

**Table 3:** rUTI patient cohort mean ± SD cytokine concentrations for IL-1, IL-5, IL-6, IL-8 and IL-10 with respect to *E. coli* carriage

Cytokine	Average Concentration		ANOVA *P*-value
	No *E. coli* (*n* = 176)	*E. coli* > 10^2^ (*n* = 184)	
IL-1	6.5 ± 14.9	8.7 ± 19.1	0.23
IL-5	12.4 ± 30.0	9.5 ± 22.8	0.30
IL-6	6.4 ± 27.9	6.3 ± 21.3	0.98
IL-8	42.9 ± 104.8	74.0 ± 348.0	0.26
IL-10	6.4 ± 12.2	9.1 ± 17.0	0.08

**Table 4:** Mean ± SD IL-10 concentrations with respect to antibiotic treatment and *E. coli* carriage

	Condition	Average IL-10 Concentration (pg/ml)	ANOVA *P*-values	
All rUTI Patients (*n* = 30)	No Antibiotics	7.7 ± 15.4	0.85	
	Antibiotics	8.0 ± 13.6		
	No *E. coli*	6.4 ± 12.2	0.56	
	< 10^5^ CFU/ml	7.3 ± 10.1		0.21^b^
	≥ 10^5^ CFU/ml	10.5 ± 20.8	0.04*	
Patients without complicated urinary tract history^a^ (*n* = 11)	No *E. coli*	3.6 ± 8.2	0.3	
	< 10^5^ CFU/ml	6.0 ± 10.3		0.05^b^
	≥ 10^5^ CFU/ml	19.3 ± 27.5	0.00002*	

* *P*-value stated is for No *E. coli* versus ≥ 10^5^ CFU/ml data sets

^a^rUTI Patients recruited to study who were not diabetic, taking oestrogen supplements, or a previous clinical history of vaginal prolapse / prostate enlargement

^b^*P*-value stated is for < 10^5^ CFU/ml versus ≥ 10^5^ CFU/ml data sets

*E. coli* persistence and infection have been reported to be more common in patients presenting with diabetes and/or a clinical history of bladder/kidney surgery [2]. When rUTI patients with diabetes, taking oestrogen supplements, or a history of vaginal prolapse, or prostate enlargement (*n* = 19), were excluded from the analyses the IL-10 data relating to *E. coli* loads ≥10^5^ CFU/ml remained significant (*P* = 0.00002) (Fig. 4c and d, Table 4).

**Fig. 5:**
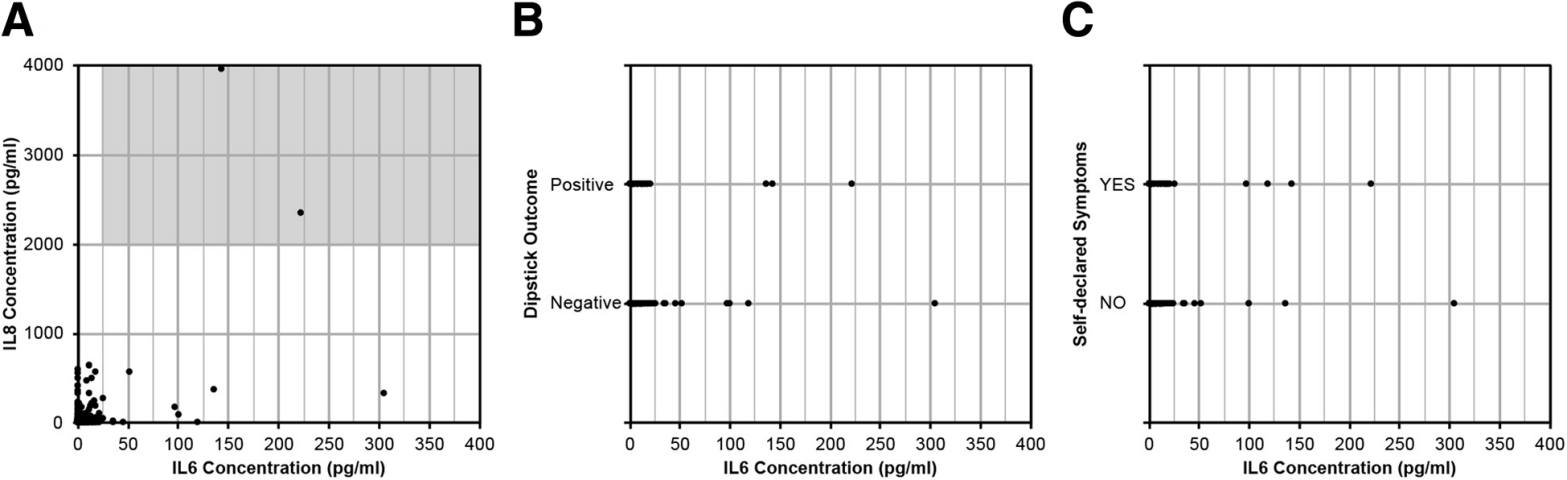
Urine concentrations of IL-6 in relation to **a** IL-8, **b** dipstick outcome and **c** self-declared symptoms. The highlighted region in (**a**) represents the required threshold for an acute UTI diagnosis based on IL-6 (> 25 pg/ml) and IL-8 (> 2000 pg/ml) as defined by Sunden et al. (2017). Both points shown in (**a**) are associated with patient UTI337 shown in Fig. 2c

IL-6 has been nominated as a biomarker in older patients that discriminates ASB from symptomatic infection [15–17]. Threshold IL-6 concentrations indicative of an UTI have been proposed as > 25 pg/ml [15] or > 30 pg/ml [17]. Applying a cut-off of > 25 pg/ml the rUTI patient IL-6 urine data identified only 13/360 measurements (Fig. 5) indicative of an UTI although only seven of these values were associated with *E. coli* counts of ≥10^5^ CFU/ml urine. Analyses of the IL-6 urine concentrations in conjunction with either IL-8 concentrations, positive dipstick (leukocyte esterase) or symptomatic data (Fig. 5a - c) revealed trends, but there was no consistency between these three sets of sample data that clarified the ASB / UTI diagnosis. However, it was noted that the two urine samples characterised by elevated IL-6 and IL-8 concentrations (Fig. 5a) also correlated with a positive dipstick outcome and the patient in question informing the study of suspected UTI symptoms.

## Discussion

Asymptomatic bacteriuria (ASB) and UTIs are common in older people, yet non-specific symptoms, often compounded by cognitive problems and the lack of good diagnostic tools to discriminate between the two conditions, can compromise the clinical management of such patients [18]. This frequently results in cautious, but un-necessary treatment regimens that achieve little clinically and challenge good antibiotic stewardship. Focussing on an older, yet cognitively sound, mixed-sex community-based population of 65+ years, and existing non-invasive methods of diagnosing an UTI, we similarly found poor concordance between self-reported patient symptoms, dip-stick, urinary cytokine and microbiological measurements. Complicating the diagnosis was the constant presence and variable numbers of bacteria in the urine of these patients. While bacteriuria is well-known to affect many older and indeed younger patient groups there is still a lack of understanding of its pathology, which continues to compromise the ASB / UTI diagnosis [7]. Therefore, to progress diagnosis and treatments for rUTI in older patients the physiological, microbiological and immunological mechanisms underpinning their bacteriuria need to be explored and unravelled.

Taking a more general approach we analysed our six-month patient and control cytokine data longitudinally, which identified the mean concentrations of four urine markers IL-1β, IL-8, IL-5 and IL-10 to be elevated within the ASB / rUTI susceptible patient cohort. Interestingly, these four molecules encompass two pro-inflammatory cytokines, IL-1β and IL-8, and two anti-inflammatory cytokines, IL-5 and IL-10 (Fig. 3), which suggested the host innate response of this older ASB / rUTI cohort had specifically adapted to tolerate urinary *E. coli* colonisation. This was further supported by the longitudinal analyses revealing cytokine responses to be maintained during periods of intermittent colonisation which were linked often, but not always, to antibiotic treatment (Table 2). However, a study limitation was that we focussed specifically on *E. coli*, therefore it was possible that other bacterial species were present and similarly impacting the host innate response.

These findings appear to suggest that in older patients with a history of ASB / rUTIs the urothelial innate defences respond and adapt to the constant microbial challenge by establishing and maintaining an urinary microbiome, defined clinically as ASB [32]. These data also suggest that this urobiome is selected and tolerated through the local production and interactions of specific host pro- and anti-inflammatory cytokines. Essentially the host creates an immune homeostasis in the lower urinary tract that supports bacterial persistence. While it could be argued that this host urobiome is a constant source of potential infection [32, 33], the counter argument is that it protects and plays a role in preventing UTIs, with misguided or precautionary short-term antibiotic use causing a dysbiosis that increases susceptibility to infection. Although our microbiological data was limited specifically to *E. coli* it was noted that some patients undergoing short-course antimicrobial treatments were characterised by *E. coli* strain shifts, albeit linked to antibiotic resistance patterns, that clinically could expose them to an increased risk of infection.

This study was designed as a pilot study with a patient cohort of 30. While the study power was justified for a pilot study [34] the small size of the patient and control cohorts was a study limitation, which arguably was further complicated by a significant difference in the average age of the female rUTI and control groups (74 compared to 68 yrs). UTI disease prevalence and pathology differ between males and females therefore comparable but larger single-sexed patient studies are needed to explore whether these IL-1β, IL-5, IL-8 and IL-10 cytokine / ASB observations are impacted by gender.

It is also acknowledged that host genetics plays a role in patient susceptibility to rUTIs [29, 35]. In children and young women (18–49 years) it has been reported that a TLR2_G2258A SNP associates with an increased risk of ASB [29, 36]. Our patient cohort size did not contain the power to examine the effects of host gene polymorphisms on microbial colonisation. However, it was noted that only 2/30 patients carried the TLR2_G2258A SNP (data not shown), suggesting that physiological or molecular events associated with ageing may either impact or supersede those due to genetics. Interestingly, age dependent differences in uropathogen susceptibility and colonisation have recently been reported in different mice strains used to model UTIs with colonisation characterised by specific cytokine / chemokine profiles [37].

While acknowledging that differences in patient genetics, lifestyle and disease pathology influence susceptibility to UTIs [29, 38, 39] it was interesting that 26 unique *E.coli* STs were identified in the rUTI patient urines with some patients, during the six-month study, carrying more than one ST. The majority of strains isolated associated with one phylogenetic clade, namely B2, but a number of STs from other clades were also identified (Fig. 2 and Table 2). Similar clade distributions of UTI isolates have been reported [40, 41] with different clade members shown to be robust (B1 and B2), deficient (A) or variable colonisers of mice bladders [41]. This study showed that antibiotic use also impacted ST prevalence but did not, however, impact cytokine production (Table 4). However, these data strongly suggest interplay between host and microbial factors, genetic and phenotypic, underpin the establishment and persistence of bacteriuria and the presentation of UTI symptoms [33].

The population targeted during this pilot study was purposely chosen to be mixed sex, aged 65+ years, susceptible to rUTIs, and community dwelling and therefore represented a relatively healthy ageing population. This could be argued as a study limitation with future studies needing also to consider other at-risk groups such as the frail, less mobile or cognitively impaired ageing individuals. A previous study in older patients, also a mix of community based males and females showing susceptibility to rUTIs, identified the urinary markers IL-6 and IL-8, both pro-inflammatory cytokines, to be elevated in bacteriuria, and significantly (4-fold) increased during acute cystitis [17]. Longitudinally the mean IL-6 urine concentration of our study participants was comparable to that of the controls (ANOVA *P* = 0.123), suggesting that this cytokine did not reflect either bacteriuria or rUTIs. However, it is acknowledged that statistical analysis of the longitudinal data may have masked elevated IL-6 values, seen as a cluster of values in Fig. 3, which reflected genuine UTIs. Further analyses (Fig. 5), did support cytokine fluctuations, but no consistent cytokine patterns were identified that specifically diagnosed an UTI. Microbiologically these urine measurements were not always associated with increased *E. coli* numbers although again it is recognised that the study focussed specifically on the association of *E. coli* with ASB although ASB can encompass a mix of organisms [7].

It was of particular interest that microbial colonisation and significant urinary *E. coli* loads in the ASB / rUTI susceptible cohort (> 10^5^ CFU/ml), were marked by the synthesis of the immunomodulatory cytokine IL-10 [42, 43]. Observations in humans and IL-10 deficient mice, support a key role for IL-10 in defending the urinary tract from UPEC infection [43, 44]. Moreover recent studies exploring UT microbial colonisation in older female mice have specifically identified IL-10 as a significant factor in their susceptibility to colonisation by *E. coli* [37]. The cytokine IL-10 is known to function in the host immune response to infectious disease [42, 43] and shown to over-ride the host inflammatory responses to infection, meaning it is closely associated with microbial persistence [45]. In the bladder markedly high IL-10 concentrations have been proposed to promote an immunosuppressive environment, which function to dampen any auto-immune responses [46] and allow the prompt regeneration of damaged epithelia [46]. Interestingly, IL-10 induction has been associated with the early phases of an infection [43] whereas our data links elevated IL-10 levels to long-term colonisation / persistence. Further evidence, albeit from young mouse UTI models, has suggested that IL-10 is synthesised primarily by migrating mast cells (MC) [27, 46] with bacterial persistence associated with elevated bladder MC numbers [46]. At present how IL-10 functions in the susceptibility and / or protection of these older patients in relation to urinary tract microbial colonisation is not known, but data does suggest a key role for the innate host response involving IL-10 in promoting bacterial persistence and potentially tolerance.

## Conclusion

Our longitudinal cytokine data from a pilot study involving 65+ year old male and female patients susceptible to ASB / rUTIs suggest that the urinary tract innate response of this ageing population has adapted to create an immune homeostasis with the IL-1β, IL-5, IL-8 and IL-10 cytokine profiles, playing a key role in the pathology of bacterial colonisation and persistence. Further patient studies are needed to confirm these observations, understand the local immune changes, particularly those involving IL-10 that underpin ABU and accompany the progression to UTI, and the impacts of therapies including antibiotics.

## Methods

### Study design

Patients were recruited with written informed consent through UTI clinics led by the Urology Department at Freeman Hospital, Newcastle upon Tyne, UK. Inclusion criteria for patients aged 65 or over with rUTI were: 1) two or more symptomatic UTIs within the last 6 months or three or more within 12 months; 2) UTIs supported clinically by urine samples positive for bacteria; 3) each diagnosed UTI associated with antibiotic treatment. Males and females were eligible, but exclusion criteria included urinary tract reconstruction, history of bladder cancer, presence of indwelling catheter or need for intermittent self-catheterisation, all of which related to a complicated UTI. Each patient in the rUTI cohort was studied for 6 months and agreed to supply a mid-stream catch urine sample every 14 days (12 samples/patient). With each urine sample the patients provided answers to a series of previously validated questions relating to their UTI status [20]. No clinical decisions were made by the research team. All rUTI patients were encouraged to seek assessment from their regular health care provider hence no urines were provided at the time points when participants approached their health-care provider in relation to an UTI. Instead clinical records were used to record the incidence of an UTI +/− 3 days of a study sample being provided and any UTI specific antibiotics prescribed.

An ethical amendment was approved in March 2016 to recruit control volunteers aged 65 years or over, with no previous history of rUTI. Male and females were enrolled utilising the volunteer network VoiceNorth (http://www.voice-global.org/) and supplied three mid-stream catch urine samples at 14-day intervals. Inclusion criteria stipulated no UTI history for the preceding 3 years which participants confirmed verbally. Screening of all urine samples, from rUTI and control cohorts, was performed in an unbiased manner. Study data was only analysed when all the urine samples had been collected and analysed.

### Analyses of urine

Urine samples from patients and controls were analysed within 4 h of collection for nitrite and leukocyte esterase using Multistix® 10SG (Siemens) according to the manufacturer’s instructions. Urines with positive nitrite and leukocyte results, were regarded as having urinalysis suggestive of an UTI. Semi-quantitative urine culture was performed according to current UK National Standard Methods of Investigation, using both 1 μl and 10 μl aliquots. Urine samples were plated onto CPS ID 3 (CPS3) or CPS Elite chromogenic agar plates (bioMérieux), incubated at 37 °C, in room air for 18–24 h. The presence of presumptive *E. coli* was noted, and the bacterial counts enumerated. The remaining urine was filtered using a 0.45 μm filter (GE Healthcare Life Sciences), and aliquots stored at − 80 °C for cytokine analyses.

Single colony isolates of *E. coli* were typed using a multi-locus sequence typing scheme [23]. Genomic DNA was isolated using the standard procedure from the Bacterial Genome Kit (Sigma). PCR products generated using published primers, at the recommended temperatures, were purified using commercial PCR Purification kits (Sigma) prior to sequencing (SourceBioscience). Sequence results were processed using the pubmlst.org website by choosing the Achtman database to identify the allele number of each gene and the corresponding sequence type.

### ELISA based cytokine detection

Ready-Set-Go!® ELISA kits (Affymetrix eBioscience) were used for the cytokine assays, which avoided frequent freeze-thawing of urine samples. All ELISAs were completed in Nunc MaxiSorp® flat-bottom 96 well plates (Affymetrix eBioscience) and the manufacturer’s protocol was followed for each of the kits. Plates were quantified at 450 and 571 nm using an Infinite® F50 / Robotic Absorbance Microplate Reader (Tecan Trading AG, Switzerland).

### Statistical analysis and data presentation

Figure generation and analysis for statistical significance was performed using a combination of R, Microsoft Excel, StatPlus and Adobe Illustrator. Statistical significance throughout this study was determined using ANOVA (Analysis of Variance). *P*-values quoted represent the actual *P*-value of the returned *F*-value generated by ANOVA [47].

## Data Availability

The datasets generated and/or analysed during the current study are not publicly available due to privacy reasons but are available in anonymized form from the authors on reasonable request.

## Abbreviations

ANOVA: Analysis of Variance
ASB: Asymptomatic bacteriuria
CFU: Colony forming units
ELISA: Enzyme linked immunosorbent Assay
MC: Mast cells
PCR: Polymerase chain reaction
rUTI: recurrent UTI
UPEC: Uropathogenic *Escherichia coli*
UT: Urinary tract
UTI: Urinary tract infections

## References

[1] Turner D, Little P, Raftery J, Turner S, Smith H, Rumsby K (2010). Cost effectiveness of management strategies for urinary tract infections: results from randomised controlled trial. BMJ.

[2] Hooton TM (2012). Uncomplicated urinary tract infection. N Engl J Med.

[3] Flores-Mireles AL, Walker JN, Caparon M, Hultgren SJ (2015). Urinary tract infections: epidemiology, mechanisms of infection andtreatment options. Nat Rev Micro.

[4] Fisher H, Oluboyede Y, Chadwick T, Abdel-Fattah M, Brennand C, Fader M (2018). Continuous low-dose antibiotic prophylaxis for adults with repeated urinary tract infections (AnTIC): a randomised, open-label trial. Lancet Infect Dis.

[5] Pulcini C, Tebano G, Mutters NT, Tacconelli E, Cambau E, Kahlmeter G (2017). Selective reporting of antibiotic susceptibility test results in European countries: an ESCMID cross-sectional survey. Int J Antimicrob Agents.

[6] Tinelli M, Cataldo MA, Mantengoli E, Cadeddu C, Cunietti E, Luzzaro F (2012). Epidemiology and genetic characteristics of extended-spectrum β-lactamase-producing Gram-negative bacteria causing urinary tract infections in long-term care facilities. J Antimicrob Chemother.

[7] Ipe DS, Sundac L, Benjamin WH, Moore KH, Ulett GC (2013). Asymptomatic bacteriuria: prevalence rates of causal microorganisms, etiology of infection in different patient populations, and recent advances in molecular detection. FEMS Microbiol Lett.

[8] Vejborg RM, Hancock V, Schembri MA, Klemm P (2011). Comparative genomics of *Escherichia coli* strains causing urinary tract infections. Appl Environ Microbiol.

[9] Zdziarski J, Svanborg C, Wullt B, Hacker J, Dobrindt U (2008). Molecular basis of commensalism in the urinary tract: low virulence or virulence attenuation?. Infect Immun.

[10] Nicolle L (2016). The paradigm shift to non-treatment of asymptomatic bacteriuria. Pathogens.

[11] Dasgupta M, Brymer C, Elsayed S (2017). Treatment of asymptomatic UTI in older delirious medical in-patients_ A prospective cohort study. Arch Gerontol Geriatr.

[12] Gharbi M, Drysdale JH, Lishman H, Goudie R, Molokhia M, Johnson AP (2019). Antibiotic management of urinary tract infection in elderly patients in primary care and its association with bloodstream infections and all cause mortality: population based cohort study. BMJ.

[13] Ali ASM, Townes CL, Hall J, Pickard RS (2009). Maintaining a sterile urinary tract: the role of antimicrobial peptides. J Urol.

[14] Sivick KE, Mobley HLT (2010). Waging war against uropathogenic *Escherichia coli*: winning back the urinary tract. Infect Immun.

[15] Sundén F, Butler D, Wullt B (2017). Triggered urine Interleukin-6 correlates to severity of symptoms in nonfebrile lower urinary tract infections. J Urol.

[16] Kjölvmark C, Tschernij E, Öberg J, Påhlman L, Linder A, Åkesson P (2016). Distinguishing asymptomatic bacteriuria from urinary tract infection in the elderly - the use of urine levels of heparin-binding protein and interleukin-6. Diagn Microbiol Infect Dis.

[17] Rodhe N, Löfgren S, Strindhall J, Matussek A, Mölstad S (2009). Cytokines in urine in elderly subjects with acute cystitis and asymptomatic bacteriuria. Scand J Prim Health Care.

[18] Rowe TA, Juthani-Mehta M (2014). Diagnosis and management of urinary tract infection in older adults. Infect Dis Clin North Am.

[19] Bardsley A (2017). Diagnosis, prevention and treatment of urinary tract infections in older people. Nurs Older People.

[20] Clayson D, Wild D, Doll H, Keating K, Gondek K (2005). Validation of a patient-administered questionnaire to measure the severity and bothersomeness of lower urinary tract symptoms in uncomplicated urinary tract infection (UTI): the UTI symptom assessment questionnaire. BJU Int.

[21] Chaudhuri RR, Henderson IR (2012). The evolution of the Escherichia coli phylogeny. Infect Genet Evol.

[22] Kaper JB, Nataro JP, Mobley HL (2004). Pathogenic *Escherichia coli*. Nat Rev Micro.

[23] Wirth T, Falush D, Lan R, Colles F, Mensa P, Wieler LH (2006). Sex and virulence in *Escherichia coli*: an evolutionary perspective. Mol Microbiol.

[24] Furuta A, Yamamoto T, Suzuki Y, Gotoh M, Egawa S, Yoshimura N (2018). Comparison of inflammatory urine markers in patients with interstitial cystitis and overactive bladder. Int Urogynecol J.

[25] Davidoff R, Yamaguchi R, Leach GE, Park E, Lad PM (1997). Multiple urinary cytokine levels of bacterial cystitis. J Urol.

[26] Bouchelouche K, Alvarez S, Horn T, Nordling J, Bouchelouche P (2006). Human detrusor smooth muscle cells release interleukin-6, interleukin-8, and RANTES in response to proinflammatory cytokines interleukin-1β and tumor necrosis factor-α. Urology.

[27] Chan CY, John ALS, Abraham SN (2013). Mast cell Interleukin-10 drives localized tolerance in chronic bladder infection. Immunity.

[28] Czaja CA, Stamm WE, Stapleton AE, Roberts PL, Hawn TR, Scholes D (2009). Prospective cohort study of microbial and inflammatory events immediately preceding *Escherichia coli* recurrent urinary tract infection in women. J Infect Dis.

[29] Hawn TR, Scholes D, Wang H, Li SS, Stapleton AE, Janer M (2009). Genetic variation of the human urinary tract innate immune response and asymptomatic bacteriuria in women. PLoS One.

[30] Köves B, Salvador E, Grönberg-Hernández J, Zdziarski J, Wullt B, Svanborg C (2014). Rare emergence of symptoms during long-term asymptomatic *Escherichia coli* 83972 carriage without an altered virulence factor repertoire. J Urol.

[31] Scottish Intercollegiate Guidelines Network (SIGN) (2012). SIGN 88 • management of suspected bacterial urinary tract infection in adults.

[32] Wolfe AJ, Brubaker L (2019). Urobiome updates: advances in urinary microbiome research. Nat Rev Urol.

[33] Ipe DS, Horton E, Ulett GC (2016). The basics of bacteriuria: strategies of microbes for persistence in urine. Front Cell Infect Microbiol.

[34] Lenth RV (2001). Some practical guidelines for effective sample size determination. Am Stat.

[35] Ali ASM, Mowbray C, Lanz M, Stanton A, Bowen S, Varley CL (2017). Targeting deficiencies in the TLR5 mediated vaginal response to treat female recurrent urinary tract infection. Sci Rep.

[36] Tabel Y, Berdeli A, Mir S (2007). Association of TLR2 gene Arg753Gln polymorphism with urinary tract infection in children. Int J Immunogenet.

[37] Armbruster CE, Smith SN, Mody L, Mobley HLT. Urine cytokine and chemokine levels predict urinary tract infection severity independent of uropathogen, urine bacterial burden, host genetics, and host age. Infect Immun. 2018. 10.1128/IAI.00327-18. 10.1128/IAI.00327-18 PMC6105902 29891542

[38] Foxman B (2002). Epidemiology of urinary tract infections: incidence, morbidity, and economic costs. Am J Med.

[39] Rowe TA, Juthani-Mehta M (2013). Urinary tract infection in older adults. Aging Health.

[40] Bielecki P, Muthukumarasamy U, Eckweiler D, Bielecka A, Pohl S, Schanz A (2014). In vivo mRNA profiling of uropathogenic Escherichia coli from diverse phylogroups reveals common and group-specific gene expression profiles. MBio.

[41] Schreiber HL, Conover MS, Chou W-C, Hibbing ME, Manson AL, Dodson KW (2017). Bacterial virulence phenotypes of *Escherichia coli* and host susceptibility determine risk for urinary tract infections. Sci Transl Med.

[42] Choi H, Abraham S (2016). Why serological responses during cystitis are limited. Pathogens.

[43] Duell BL, Tan CK, Carey AJ, Wu F, Cripps AW, Ulett GC (2012). Recent insights into microbial triggers of interleukin-10 production in the host and the impact on infectious disease pathogenesis: Table 1. FEMS Immunol Med Microbiol.

[44] Sundac L, Dando SJ, Sullivan MJ, Derrington P, Gerrard J, Ulett GC (2016). Protein-based profiling of the immune response to uropathogenic Escherichia coliin adult patients immediately following hospital admission for acute cystitis. Pathog Dis.

[45] Mege J-L, Meghari S, Honstettre A, Capo C, Raoult D (2006). The two faces of interleukin 10 in human infectious diseases. Lancet Infect Dis.

[46] Choi HW, Bowen SE, Miao Y, Chan CY, Miao EA, Abrink M (2016). Loss of bladder epithelium induced by cytolytic mast cell granules. Immunity.

[47] Aldridge P, Karlinsey J, Hughes KT (2003). The type III secretion chaperone FlgN regulates flagellar assembly via a negative feedback loop containing its chaperone substrates FlgK and FlgL. Mol Microbiol.

